# Roles of parents in life satisfaction and educational hope among Chinese high school students

**DOI:** 10.3389/fpsyg.2024.1334397

**Published:** 2024-04-15

**Authors:** Feng Han, Celeste Yuet-Mui Yuen

**Affiliations:** ^1^Institute of Education, Tsinghua University, Beijing, China; ^2^Department of Educational Administration and Policy, Faculty of Education, Hong Kong Centre for the Development of Educational Leadership, The Chinese University of Hong Kong, Shatin, Hong Kong SAR, China

**Keywords:** life satisfaction, hope, educational aspirations, goal commitment, parental support, *gaokao*, high school students

## Abstract

The well-being of the Chinese high school students linked to the National Higher Education Entrance Examination, known as gaokao, has been a spotlight education issue in China. This study employed self-determination theory and Bourdieu’s sociocultural theory to examine the relationship between life satisfaction, educational hope, and parental support among Chinese high school students. A number of 3,810 high school students from eight schools in Jiangsu, China, completed a validated context-relevant questionnaire. Structural equation model analysis suggested that parental support significantly impacted students’ life satisfaction and educational hope. Findings showed that parental intangible support in terms of providing information, advice, encouragement, praise, and care has a direct and significant impact on the life satisfaction of Chinese youth. The extent to which students attach importance to and put effort into achieving their educational aspirations, known as goal commitment, mediated the relationship between parental support and life satisfaction. Moreover, Chinese high school students’ educational hope is shaped by their family. Parental support moderates goal commitment, which varies based on parental education background. In short, parents play a critical role in the growth and development of Chinese high school students.

## 1 Introduction

Mainland Chinese students must pass the high-stake and highly competitive National Higher Education Entrance Examination, known as the *gaokao* in Chinese, to enter university. High school is a critical stage for Chinese youth as it involves preparations for *gaokao*: students typically begin preparing for the Gaokao starting from the 9th grade (around 15–16 years old), and the pressure they face for the gaokao increases as they advance in academic grades (Pang and Zhang, 2021). The legacy of the Confucian examination culture in China upholds the selection mechanism of *gaokao* (Yi, 2012), which is the most widely regarded public examination for higher education stratification. Up to 10 million students take the *gaokao* (China Education Online, 2019) yearly; the results always make headlines. Thus, education aspirations are nurtured by families and reinforced by schools and social values.

Undoubtedly, *gaokao* intensifies the stress of transitioning from high school to university among Chinese high school students and causes their overall health levels to be lower than those of the general population (Ji et al., 2016). Such life impediments mediate their well-being, and recent studies have revealed alarming figures of mental health issues among *gaokao* students, including anxiety (34%), depression (28%), and sleep disorders (23%) (Chen et al., 2017; Yu et al., 2022). The number of anxiety disorder cases has increased over time (Chen et al., 2017). Hence, the well-being of *gaokao* students in relation to their preparation for higher education examinations is a pressing issue that must be addressed.

Chinese high school students have a strong desire to succeed in *gaokao* reflects their educational hope. Educational hope is a combination of educational aspirations and goal commitment in education. Hope motivates young adults to set and pursue a meaningful goal, which enhances their sense of meaning in life and contributes to their subjective well-being (Guse and Shaw, 2018). Previous studies have confirmed that hope positively predicts academic performance and subjective well-being (Griggs and Crawford, 2017; Dixson, 2019; Pleeging et al., 2021). However, little is known about how educational hope promotes young people’s subjective well-being and how different aspects of educational hope, such as educational aspirations and goal commitment, are related to their subjective well-being.

Recent research has emphasized the crucial role that parents play in promoting youth subjective well-being and education aspirations. Research has shown that parent–child closeness and emotional support from parents can increase youth subjective well-being (King et al., 2018; Kiuru et al., 2020). Furthermore, parents’ education and tangible support can facilitate their children’s educational aspirations through cultural and social capital (Hartas, 2016; Fischer et al., 2019; Hadjar et al., 2021; Ng and Choo, 2021). There is currently a lack of research that explores how various forms of parental support can impact their children’s educational hope and well-being. This study is a response to examine the relationships among subjective well-being, educational hope, and parental support among high school students in China, by studying how various types of parental support affect multiple aspects of educational hope and subjective well-being, as well as how educational hope relate to subjective well-being. Through this study, we hope to provide evidence-based and contextually relevant suggestions for improving the well-being of young people in China and beyond, considering the influence of the Confucian examination culture.

## 2 Literature review

### 2.1 Well-being and life satisfaction

Well-being is widely recognized as a quality of life and is embodied in an individual’s positive functioning and overall flourishing (Ryan and Deci, 2001); it has, thus, become increasingly important in youth studies and social policy as highlighted by Shenaar-Golan and Goldberg (2019). According to Noddings (2003), well-being is the ultimate goal of schooling and is closely associated with youth development. Since young people are still in the process of growing and changing, their well-being is vital for positive self-adjustment, such as the adaptation in stressed life (Lee et al., 2021) and school-related outcomes (Lewis et al., 2011), such as school engagement (Cheng et al., 2021) and academic achievement (Griggs and Crawford, 2017; Kiuru et al., 2020).

Previous research on the subjective well-being of young people has mostly focused on their life satisfaction, which pertains to their cognitive judgment of well-being (Diener et al., 2009). Researchers suggest that supportive social relationships (Koestner et al., 2020), self-esteem (Yan et al., 2021), and stressful events (Wendt et al., 2019) can all impact well-being outcomes. Studies on young people’s life satisfaction have been conducted in various contexts, but most of them have been carried out in Western countries, with limited empirical research examining the well-being of Asian youth (Yuen, 2016) and its association with academic-related variables.

### 2.2 Hope and educational hope

Hope is a state of mind in which individuals have a fervent desire to achieve and actively work toward it (Pleeging et al., 2021). This goal-oriented thinking is backed by hope theory (Snyder, 2002), which defines hope as a dynamic cognitive process that involves agency and pathway thinking (Colla et al., 2022). Hopeful individuals are committed to achieving their goals and exhibit perseverance in their pursuit. Research has shown that hope is a strong predictor of academic performance (Dixson, 2019; Rand et al., 2020), emotional health (Griggs and Crawford, 2017) and overall well-being among college students (Pleeging et al., 2021).

Educational hope refers to the hope that one has in education. It is a combination of educational aspirations and goal commitment, which are the extent to which students attach importance to educational goals and actively work toward achieving them. However, it is unclear whether educational hope affects youth subjective well-being. Some researchers have examined the relationship between educational aspirations and subjective well-being, but there is no consistency in their findings. For instance, Liu and Helwig (2022) deem that educational aspirations lead to high levels of stress and often cause students to ignore their health and intrinsic motivation, ultimately harming their well-being. On the other hand, Cao et al. (2022) found that educational aspirations have a positive impact on the well-being of young Chinese people. Meanwhile, Wang et al. (2021) argue that increasing high school students’ educational hope and motivation is a more effective way to improve their well-being scores than directly decreasing their academic stress.

### 2.3 Parents’ roles in life satisfaction and educational aspirations

Parents are the primary caregivers and educators of their children, particularly in societies where collective culture prevails (Lam et al., 2020). Family background and parental support significantly contribute to the well-being and growth of children. Research has consistently shown that family background factor such as family socio-economic status and parental education level, is a positive predictor for educational aspirations (Hadjar et al., 2021; Ng and Choo, 2021) and subjective well-being (Assari, 2018; Ge, 2020). Parental support, which refers to the supportive parent–child relationship, has been confirmed to be more influential than school-related support on the subjective well-being (Blau et al., 2019) and goal pursuit (Koestner et al., 2020) of young people.

Parental support can be categorized into four dimensions: instrumental, conditional, motivational, and informational. Instrumental support includes providing resources and practical assistance, while conditional support involves monitoring, supervision, and participation in school activities (Beets et al., 2010, P. 624). The former two types are regarded as tangible support while the latter two types are defined as intangible support. Numerous studies have shown that a positive and nurturing relationship between parents and children, where parents provide encouragement and warmth, can contribute to the overall life satisfaction of young people (Lv et al., 2016; Koestner et al., 2020). However, studies examining the impact of tangible parental support on the well-being of young people have yielded inconsistent results. For instance, Danielsen et al. (2009) suggest that tangible educational support from parents can enhance academic performance and outcomes, which, in turn, can improve life satisfaction. Conversely, Shenaar-Golan and Goldberg (2019) argue that excessive control and inadequate supervision from parents can negatively affect the life satisfaction of young people. While Podaná and Krulichová (2018) believe that parental supervision is inversely linked to youth life satisfaction. Moreover, Hartas (2016, p. 1145) pointed out that parent–child interactions in cultural events and emotional bonding are more robust predictors of children’s educational aspirations than supervision and arrangement of extracurricular activities. Therefore, further studies are required to consider the effects of different types of parental support on youth life satisfaction and educational hope.

## 3 Theoretical framework

Social capital theories have been applied not only to explain the formation of educational hope but also to illustrate the effects of parental support on well-being. These theories are useful for unraveling the relationship between parental support, educational hope, and life satisfaction. Framed by cultural and social capital theories, creating a positive family environment, including providing emotional support, consistent routines, respect, and communication at home, positively influences students’ well-being and disposition and inspires their ambitions (Bourdieu, 1977, 1986).

Furthermore, through the parent–child connection, parents activate cultural capital (Lareau, 2000) and impart their educational expectations and cultural competence to their children (Danielsen et al., 2009). In other words, as a form of social capital, parental support can convey parents’ attitudes toward education through various forms of interaction, thereby shaping their children’s educational hope. Therefore, this theory provides a foundation for understanding the relationship between parental support and educational hope. In addition, based on social capital theories, parent–child supportive relationships can be defined as family social capital for youth subjective well-being (Ge, 2020). In fact, the perspective of positive psychology can help further elucidate the mechanisms by which parental support and goal pursuit influence subjective well-being.

Self-determination theory (SDT) explains the relationships between well-being, goal pursuit and parental support. According to SDT, people’s well-being is linked to their satisfaction with their psychological needs for autonomy, competence, and relatedness (Ryan and Deci, 2017). Personal goal pursuit can contribute to well-being if these psychological needs are met during goal progress (Ryan and Deci, 2000). For instance, students feel a sense of well-being when they strive for educational aspirations that meet their competence needs. Additionally, parental support promotes well-being in youth by satisfying their need for relatedness and competence, as observed in the context of China (Yan et al., 2021).

The home advantage factor has been established by research (Lareau, 2000). Middle-class parents are able to offer expert advice on educational choices, develop strategies for navigating program pathways, and increase their children’s social networks for lifelong opportunities and economic resources. Furthermore, interactions between parents and children can be seen as beneficial social capital in the form of care and material resources that benefit the child (Coleman, 1988, 1994; Ho, 2010), cultivate their academic abilities, and, in turn, predict their life satisfaction (Danielsen et al., 2009). Therefore, this study used the level of parental education as a grouping variable to analyze the differences between groups.

**Figure 1:**
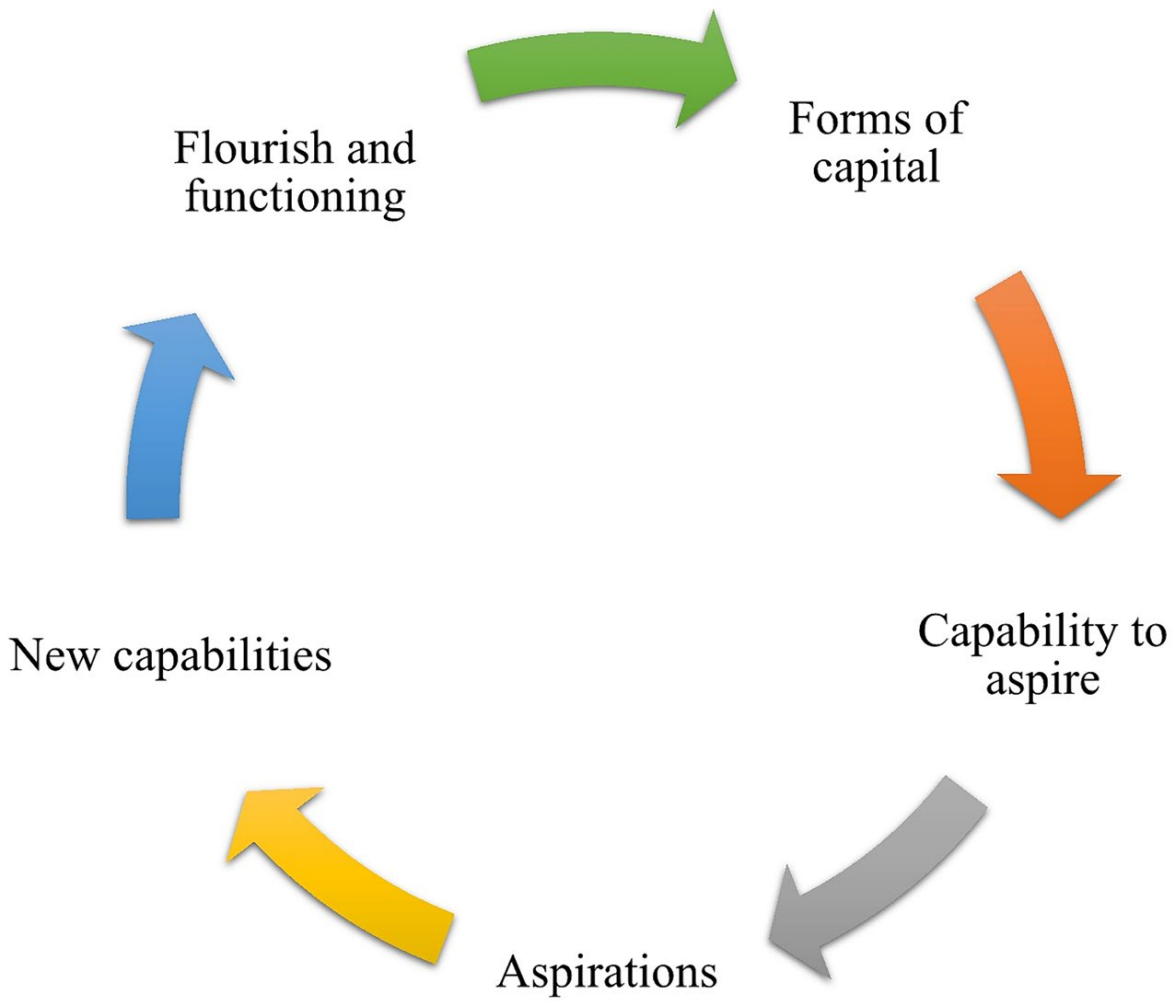
Aspiration formation and realization.

Drawing on Bourdieu’s (1977, 1986) sociocultural theories and Sen’s perspective on the capability to flourish (Sen, 1992), Hart (2012, 2016) proposed a framework that examines the relationships among parental support, aspirations, and well-being. The framework suggests that sociocultural capital plays an essential role in promoting an individual’s capability for high life aspirations, while personal life aspirations cultivate the capability that leads to flourishing (Hart, 2012, 2016). The relationships between these factors are depicted in Figure 1. Using this framework helps to consider all factors, including parental support, parental education, educational hope, and life satisfaction, in combination.

## 4 The current study and hypotheses

**Figure 2:**
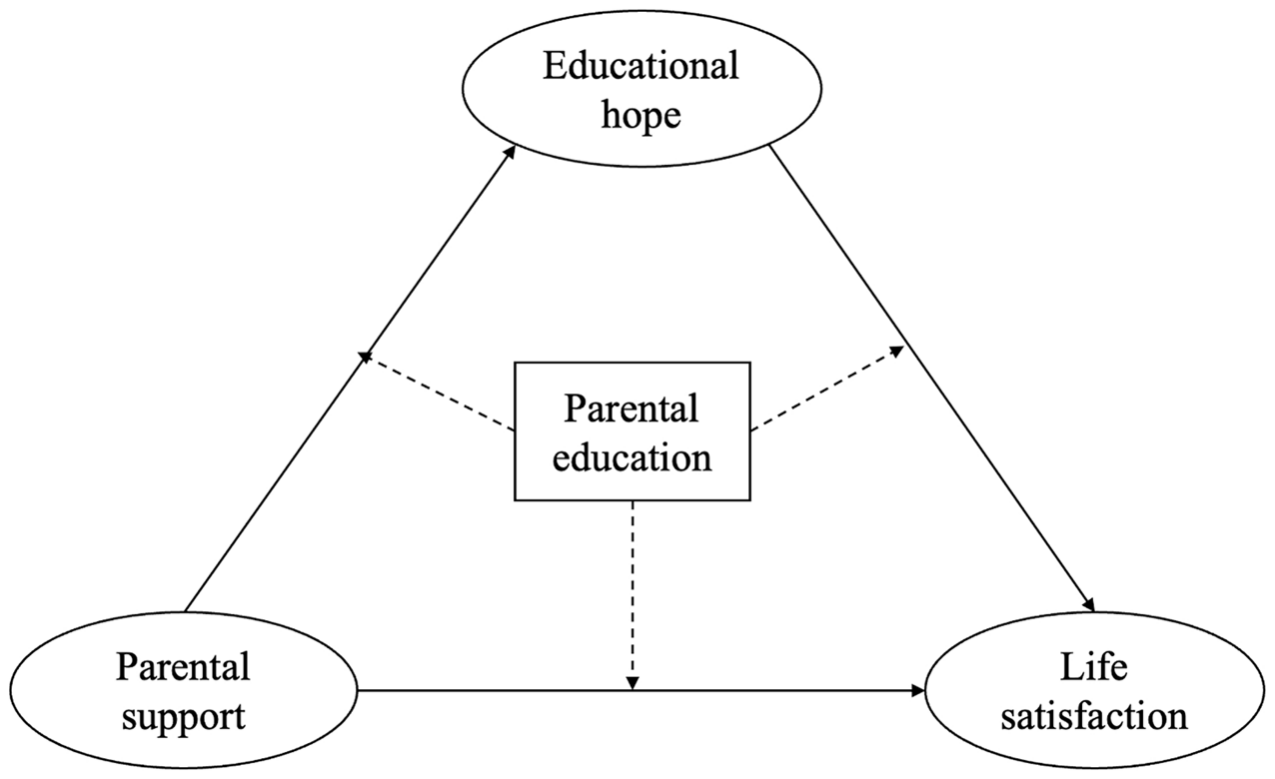
The hypothesized model. The solid lines indicate hypothesized relationships while the dotted lines indicate moderated effects.

This study explored the complex connections between life satisfaction, educational hope, and parental support among Chinese high school students. The research was based on existing literature and Hart’s (2012, 2016) framework. The capital types are classified as cultural and social capital at home, which are expressed through parental education and support in both tangible and intangible ways. Aspirations refer to educational hope, which includes educational aspirations and goal commitment, while flourishing is associated with well-being, which is assessed by life satisfaction. In line with the objectives of the present study, the following three hypotheses are proposed (see Figure 2):

*Hypothesis 1 (H1)*: Life satisfaction and educational hope are positively predicted by parental support.

*Hypothesis 2 (H2)*: Educational hope mediates the effects of parental support on life satisfaction.

*Hypothesis 3 (H3)*: Parental education moderates the interrelationships among parental support, educational hope, and life satisfaction.

## 5 Methods

### 5.1 Samples and procedure

In this study, a three-step sampling method was used. The first step was to choose Xuzhou in Jiangsu Province, China, as the case study. Jiangsu is known for being one of the most competitive provinces in *gaokao*, and previous research has discovered that high school students in East China, including Jiangsu, have the highest detection rate of anxiety disorders (Chen et al., 2017). By selecting this region, we were able to show the variable intensity (Patton, 2002). For the second step, we used a stratified purposeful sampling method to select eight high schools in Xuzhou based on the district distribution and criteria of Jiangsu’s ‘star rating system’ for high schools.

Finally, we used cluster sampling to select 96 classes from *gaokao* 1–3 (10th – 12th graders). Four classes at each grade level were randomly selected from each school. The teacher coordinators assisted us in conducting the online survey with the target participants. The research design was approved by the Survey and Behavioral Ethical Review Committee of the authors’ university.

Given that this study was conducted during the COVID-19 outbreak in China, when social distancing regulations were strictly implemented, an online questionnaire survey was administered to collect data. To ensure the accuracy of the data, a pilot test was conducted, which showed that participants took approximately 5 min on average to complete the questionnaire. Thus, during the main study, any responses that were completed in less than 5 min were considered invalid and were deleted.

The sample size after data cleaning was 3,810 students aged 16–19 years, with a valid response rate of 86.59%. 49.2% were male and 50.8% were female. Around two-thirds of the parents had no college experience (71.1%) and the rest had college experience (28.9%). Students whose parents had no college experience were considered as the reference group (Group 1).

### 5.2 Measures

Life satisfaction was measured by an adapted Multidimensional Student Life Satisfaction Scale. This scale assesses students’ satisfaction with five life domains: family, friends, school, self, and living environment (Huebner, 1994; Gilman et al., 2000). The version of the scale that was translated and adapted by Yuen and Lee (2016) and has been previously administered in Hong Kong with satisfactory validity and reliability (Yuen, 2013, 2016). To ensure the effectiveness of the responses, we simplified this version based on its structure and our exploratory factor analysis (EFA) in the pretest. The final version of the questionnaire comprised 17 items. The respondents were required to indicate their agreement with statements such as ‘I feel happy at school’ on a 6-point Likert scale ranging from 1 (strongly disagree) to 6 (strongly agree). In our study, this scale displayed satisfactory internal consistency (α = 0.947) and performed well in the confirmatory factor analysis (CFA) [χ^2^ (118) = 847.788 (*p* < 0.001), root mean square error of approximation (RMSEA) = 0.048, comparative fit-index (CFI) = 0.967, Tucker-Lewis index (TLI) = 0.962].

Educational hope is a combination of educational aspirations and goal commitment. Educational aspirations were measured by questions such as ‘What is your ultimate educational goal?’ which refers to the final education level that one wishes to achieve. Four categories of aspirations were coded based on China’s educational system as follows: (1) 12 = None or graduating from high school, (2) 15 = Attending a three-year vocational college, (3) 16 = Attending a four-year university, and (4) 19 = Obtaining a postgraduate degree. Goal commitment was measured using the five-item Hollenbeck, Williams, and Klein Goal Commitment Scale. One sample item was ‘I think this is a good goal to shoot for’ (Klein et al., 2001, p. 34). The participants reported their agreement on a 6-point Likert scale ranging from 1 (strongly disagree) to 6 (strongly agree). The items were translated into Chinese, and back-translation and verification of equivalence were performed. The validity [χ^2^ (5) = 83.615 (*p* < 0.001), RMSEA = 0.068, CFI = 0.950, TLI = 0.922] and internal reliability (α = 0.852) of this scale were satisfactory for our sample.

The academic-related support subscale in Yuen and Lee’s (2016) questionnaire was used to evaluate parental support for Hong Kong adolescents’ well-being and academic performance. In our pilot study, the EFA results identified three items as tangible parental support, such as ‘My parents discuss with me about my learning’ and ‘My parents provide resources for my learning.’ Three items were seen as intangible parental support, such as ‘My parents encourage me when I am in trouble’ and ‘My parents offer advice when I need it’. The CFA results showed that the model had an acceptable fit [χ2 (8) = 107.301 (*p* < 0.001), RMSEA = 0.063, CFI = 0.975, TLI = 0.953]. The six-item scale’s coefficient alphas were greater than 0.90 (α = 0.930), indicating satisfactory internal reliability.

### 5.3 Analytical strategies

Descriptive statistics, including *t-test* for group comparison and Pearson correlation analysis, were performed using SPSS 25.0. Structural equation modeling via Mplus 8.0. was used to test the hypotheses. Path analysis with maximum likelihood estimation was performed to examine the relationships among the key variables. Mediation and multigroup analyses were also conducted to verify the last two hypotheses. Participants were required to answer all questions to complete the online questionnaire. Accordingly, incomplete questionnaires and missing data were excluded from the analysis.

## 6 Results

### 6.1 Descriptive statistics

In the first step, descriptive statistics are reported to provide an overview of the characteristics of the key variables in this study. Table 1 shows the means, standard deviations, and *t-test* results, and Table 2 presents the correlations of the key variables between the two groups.

**Table 1:** Descriptive statistics of the main variables.

Scales/subscales	Range	Mean (*SD*)		*t*	Cohen’s *d*
		Group 1: *n* = 2,710	Group 2: *n* = 1,100		
Life satisfaction					
Family	1–6	5.223 (0.93)	5.226 (1.03)	−0.089	0.003 < 0.20
Friends	1–6	5.078 (1.01)	5.163(1.06)	−2.268^*^	0.082 < 0.20
School	1–6	4.599 (1.28)	4.734 (1.31)	−2.921^**^	0.104 < 0.20
Self	1–6	4.810 (1.15)	4.931 (1.24)	−2.901^**^	0.101 < 0.20
Living environment	1–6	4.931 (0.99)	5.148 (0.94)	−6.217^***^	0.225
Higher education aspirations					
Educational aspirations	12–19	16.724 (1.69)	17.238 (1.78)	−8.211^***^	0.297
Goal commitment	1–6	5.382 (0.74)	5.307 (0.84)	−2.573^**^	0.095 < 0.20
Parental support					
Tangible support	1–6	4.848 (1.24)	5.112 (1.13)	−6.363^***^	0.223
Intangible support	1–6	4.789 (1.12)	5.016 (1.07)	−5.849^***^	0.207

The students whose parents had no college experience were considered the reference group (Group 1). Cohen’s *d* (effect size): Substantial significance based on the *t*-test with a threshold value above 0.20. ^*^p < 0.05, ^**^p < 0.01, ^***^p < 0.001.

**Table 2:** Correlations among key variables between the two groups.

Variables	1	2	3	4	5
(1) Life satisfaction	—	0.124^***^	0.449^***^	0.532^***^	0.598^***^
(2) Educational aspirations	0.156^***^	—	0.294^***^	0.121^***^	0.192^***^
(3) Goal commitment	0.433^***^	0.343^***^	—	0.419^***^	0.490^***^
(4) Tangible support	0.501^***^	0.150^***^	0.299^***^	—	0.767^***^
(5) Intangible support	0.597^***^	0.202^***^	0.391^***^	0.696^***^	—

Correlations for Group 1 are below the diagonal while the correlations for Group 2 are above the diagonal. ^***^p < 0.001.

Life satisfaction: As shown in Table 1, the respondents were moderately satisfied with their lives, with the levels of the five dimensions ranging from 4.5 to 5.3 on a 6-point Likert scale, consistent with previous Chinese findings (Tian and Liu, 2005). The reference group exhibited significantly lower levels of life satisfaction. However, the reference group, whose parents had no college experience, exhibited significantly lower level of life satisfaction. They were less satisfied with their friends (mean = 5.078), their school (mean = 4.599), themselves (mean = 4.810), and their living environment (mean = 4.931) than their counterparts (mean = 5.162, 4.734, 4.931, 5.148). The differences in satisfaction with the living environment between the two groups were substantial, with Cohen’s *d* between 0.2 and 0.5 (*p* < 0.001, Cohen’s *d* = 0.225).

Educational hope: The vast majority of participants (97.2%) expressed a desire to attend college, while 88.4% aimed for a university education or higher. On average, participants aspired to obtain a university education, with the means of educational aspirations slightly above 16 (as shown in Table 1). Those whose parents had a college background reported higher levels of educational aspirations than their counterparts (mean difference = 0.514, *p* < 0.001, Cohen’s *d* = 0.297), which is consistent with previous research (Guo et al., 2015). Additionally, participants with a high level of goal commitment (Group 1: mean = 5.382; Group 2: mean = 5.307) exhibited a strong determination to pursue a university education.

Parental support: Table 1 presents the data on respondents who reported moderately strong levels of tangible and intangible parental support (Group 1: mean = 4.848; Group 2: mean = 5.112 for tangible support, and Group 1: mean = 4.789; Group 2: mean = 5.016 for intangible support). The reference group had lower levels of both tangible support (mean = 4.848) and intangible support (mean = 4.789) compared to their counterparts (mean = 5.112, 5.016) with a statistically significant difference (*p* < 0.001, Cohen’s *d* = > 0.20). This result confirms the cultural capital view that students with highly educated parents benefit more from their parents’ support (Lareau, 2000).

In Table 2, the variables related to parental support were positively correlated with educational hope and life satisfaction. The correlation between life satisfaction, goal aspiration, and goal commitment was weak (Group 1: *r* = 0.343; Group 2: *r* = 0.294).

### 6.2 Path analysis

Path analysis examined the relationships between life satisfaction, educational hope, and parental support. The results indicated that tangible and intangible parental support contributed significantly to life satisfaction. Notably, intangible parental support significantly predicted educational hope (educational aspirations and commitment) and consequently predicted life satisfaction positively through goal commitment. Thus, the first hypothesis was confirmed.

Furthermore, the structural relationships among the variables confirmed that the hypothesized model was acceptable with a reasonable fit [Group 1, χ^2^ (445) = 2238.079 (*p* < 0.001), RMSEA = 0.055, SRMR = 0.050, CFI = 0.944, TLI = 0.937; Group 2, χ^2^ (445) = 1333.373 (*p* < 0.001), RMSEA = 0.060, SRMR = 0.063, CFI = 0.935, TLI = 0.927].

Table 3 presents the unstandardized path coefficients (B), standardized path coefficients (*β*), significance, and effect sizes of each path. The total and direct effects of parental support on life satisfaction were positive and significant. Intangible parental support had a significantly positive effect on educational hope.

**Table 3:** Path analysis results.

Path	*B*	S.E.	*β*	C.R.	*p-*value	*f* ^2^
Group 1, *n* = 2,710						
Direct effects						
Tangible support → Life satisfaction	0.223	0.049	0.184	4.668	<0.001	0.046
Intangible support → Life satisfaction	0.733	0.091	0.452	9.664	<0.001	0.204
Tangible support → Educational aspirations	−0.039	0.057	−0.029	−0.679	>0.05	0.000
Intangible support → Educational aspirations	0.386	0.085	0.215	4.628	<0.001	0.023
Tangible support → Goal commitment	0.015	0.024	0.031	0.642	>0.05	0.003
Intangible support → Goal commitment	0.272	0.037	0.408	8.172	<0.001	0.098
Educational aspirations → Life satisfaction	−0.038	0.023	−0.042	−1.680	>0.05	0.002
Goal commitment → Life satisfaction	0.670	0.106	0.275	7.408	<0.001	0.128
Indirect effects						
Tangible support → Educational aspirations → Life satisfaction	0.001	0.003	0.001	0.555	>0.05	
Tangible support → Goal commitment → Life satisfaction	0.010	0.016	0.008	0.637	>0.05	
Intangible support → Educational aspirations → Life satisfaction	−0.015	0.010	−0.009	−1.544	>0.05	
Intangible support → Goal commitment → Life satisfaction	0.182	0.034	0.112	5.666	<0.001	
Total effects						
Tangible support → Life satisfaction	0.235	0.051	0.193	4.695	<0.001	
Intangible support → Life satisfaction	0.900	0.094	0.555	12.464	<0.001	
Group 2, *n* = 1,100						
Direct effects						
Tangible support → Life satisfaction	0.234	0.113	0.190	2.180	<0.05	0.032
Intangible support → Life satisfaction	0.494	0.180	0.341	3.040	<0.01	0.075
Tangible support → Educational aspirations	−0.122	0.128	−0.076	−0.960	>0.05	0.003
Intangible support → Educational aspirations	0.467	0.162	0.247	2.967	<0.01	0.026
Tangible support → Goal commitment	0.019	0.056	0.032	0.341	>0.05	0.006
Intangible support → Goal commitment	0.362	0.070	0.512	5.838	<0.001	0.152
Educational aspirations → Life satisfaction	−0.091	0.030	−0.119	−3.054	<0.01	0.022
Goal commitment → Life satisfaction	0.550	0.142	0.269	4.082	<0.001	0.083
Indirect effects						
Tangible support → Educational aspirations → Life satisfaction	0.011	0.014	0.009	0.836	>0.05	
Tangible support → Goal commitment → Life satisfaction	0.011	0.032	0.009	0.341	>0.05	
Intangible support → Educational aspirations → Life satisfaction	−0.042	0.022	−0.029	−1.958	>0.05	
Intangible support → Goal commitment → Life satisfaction	0.199	0.056	0.138	3.671	<0.001	
Total effects						
Tangible support → Life satisfaction	0.256	0.120	0.208	2.240	<0.05	
Intangible support → Life satisfaction	0.651	0.167	0.450	4.538	<0.001	

*f*^2^ (effect size) – cut-off values: 0.02 < *f*^2^ < 0.15 (small), 0.15 < *f*^2^ < 0.35 (medium), *f*^2^ > 0.35 (large).

**Figure 3:**
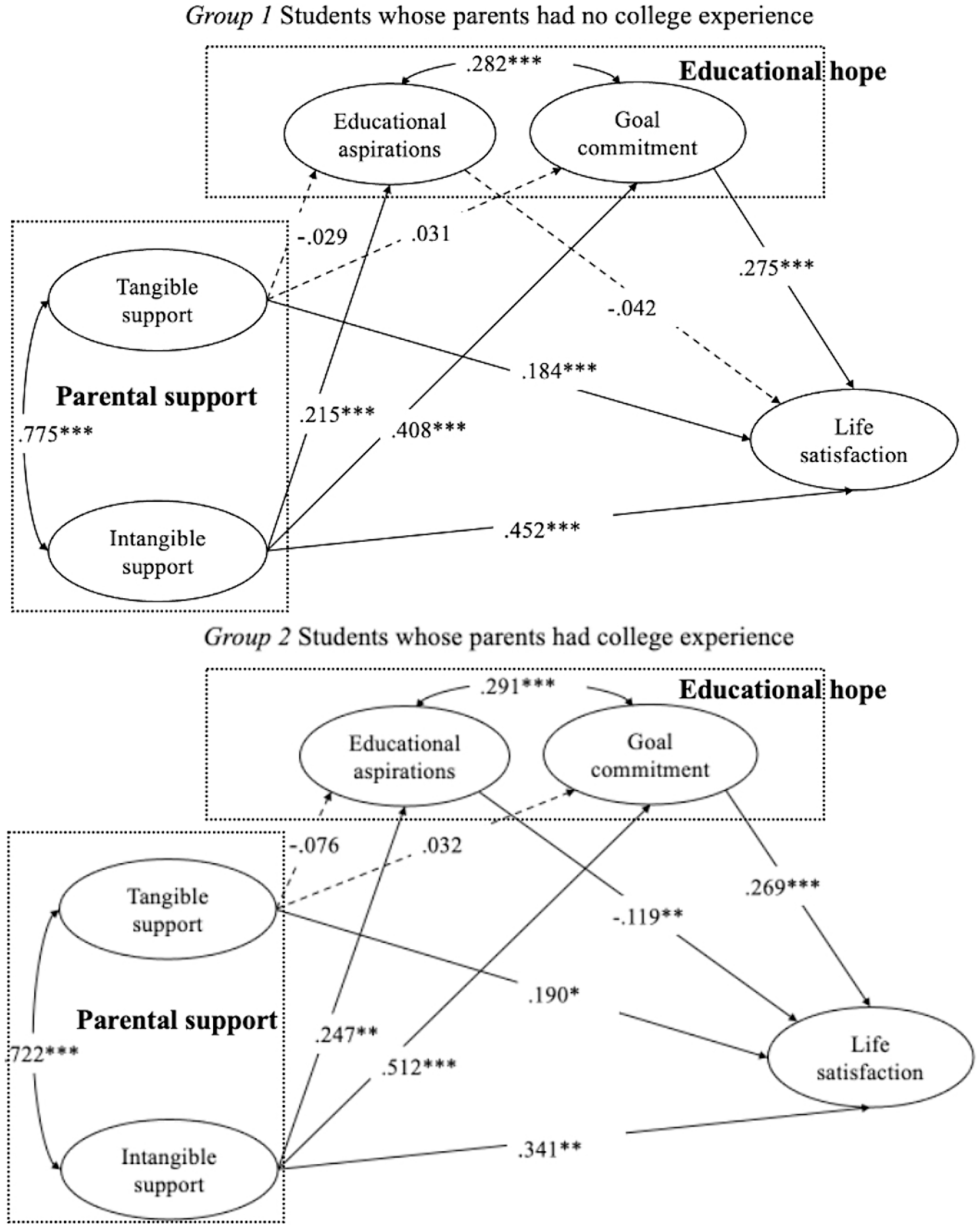
Structural equation models for the two groups. Solid lines indicate significant relationships; dotted lines show non-significant relationships. ^*^p < 0.05, ^**^p < 0.01, ^***^p < 0.001.

Figure 3 presents the standardized path diagram with *β across two groups*. (1) Life satisfaction was directly and positively predicted by tangible parental support (Group 1: *β* = 0.184, *p* < 0.001; Group 2: *β* = 0.190, *p* < 0.05) and intangible parental support (Group 1: *β* = 0.452, *p* < 0.001; Group 2: *β* = 0.341, *p* < 0.01). (2) Educational aspirations (Group 1: *β* = 0.215, *p* < 0.001; Group 2: *β* = 0.247, *p* < 0.01) and goal commitment (Group 1: *β* = 0.408, *p* < 0.001; Group 2: *β* = 0.512, *p* < 0.01) were positively predicted by intangible parental support. (3) In the same vein, intangible parental support indirectly and positively predicted life satisfaction via goal commitment (Group 1: *β* = 0.112, *p* < 0.001; Group 2: *β* = 0.138, *p* < 0.001). These results reveal that Chinese high school students’ high value for *gaokao* and their high engagement benefit their life satisfaction. Moreover, intangible parental support plays a greater role in students’ life satisfaction than tangible parental support. These results provide the prerequisites for mediation analysis, as described in the following section.

### 6.3 Mediating effects of educational hope on parental support and life satisfaction

The path analysis revealed that intangible parental support played a significant role in improving goal commitment and life satisfaction. Three prerequisites for mediation effects were confirmed as follows: (1) a significant total effect of the independent variable (intangible parental support) on the dependent variable (life satisfaction), (2) a significant direct effect of the independent variable on the mediator (goal commitment), and (3) a significant direct effect of the mediator on the dependent variable. To test the second hypothesis, bootstrapping approaches and a 95% confidence interval were used to examine the significance of the mediation of goal commitment on intangible parental support and life satisfaction. The study yielded significant results that suggested the estimated mediation path was significant for both Group 1 (*β* = 0.112, *p* < 0.001, CR = 5.666, 95% CI = 0.081–0.134) and Group 2 (*β* = 0.138, *p* < 0.001, CR = 3.671, 95% CI = 0.085–0.205). This means that goal commitment partially mediated the relationship between intangible parental support and life satisfaction. Therefore, the second hypothesis was supported.

### 6.4 Multigroup comparison of parents with and without higher education backgrounds

We performed a multigroup analysis to compare group differences in path coefficients, following the method outlined by Preacher et al. (2007). Our objective was to examine the moderating effects of parental educational background on life satisfaction, educational hope, and parental support. We first tested a baseline model where all path coefficients were freely estimated across two groups: Group 1, consisting of students whose parents had no higher education backgrounds, and Group 2, consisting of students whose parents had higher educational backgrounds. We then assessed a series of constrained models and compared the differences in χ^2^ (increment 2) between the baseline and constrained models, as described in Hau et al. (2004). As shown in Table 4, we found that χ^2^ significantly increased when we constrained the path from intangible parental support to goal commitment (*p* < 0.01). As shown in Table 2 and Figure 3, the effect of intangible parental support on goal commitment was weaker for Group 1 (*B* = 0.272, *β* = 0.408, *f*2 = 0.098) than it was for Group 2 (*B* = 0.362, *β* = 0.512, *f*2 = 0.152). In summary, we found that parental educational backgrounds moderated the relationship between these two variables, supporting our third hypothesis.

**Table 4:** Model fit comparisons across groups.

Model		χ^2^	*df*	RMSEA	SRMR	CFI	TLI	� χ^2^
Baseline model		3,744.312	899	0.058	0.056	0.937	0.931	
Constrained models								
1	‘Tangible parental support → Life satisfaction’ was constrained	3,746.754	900	0.058	0.056	0.937	0.931	2.442
2	‘Intangible parental support → Life satisfaction’ was constrained	3,745.783	900	0.058	0.057	0.937	0.931	1.471
3	‘Intangible parental support → Educational aspirations’ was constrained	3,745.152	900	0.058	0.056	0.937	0.931	0.840
4	‘Intangible parental support → Goal commitment’ was constrained	3,752.606	900	0.058	0.057	0.937	0.931	8.294^**^
5	‘Educational aspirations → Life satisfaction’ was constrained	3,746.303	900	0.058	0.056	0.937	0.931	1.991
6	‘Goal commitment → Life satisfaction’ was constrained	3,745.889	900	0.058	0.056	0.937	0.931	1.577

When Δdf = 1, if Δχ^2^ > 3.84, *p* < 0.05; if Δχ^2^ > 6.63, *p* < 0.01, ^*^p < 0.05, ^**^p < 0.01.

## 7 Discussion

### 7.1 The influences of parental intangible support

The results of this study offer fresh insights into the intricate connections between parental support, educational hope (educational aspirations and goal commitment), and the life satisfaction of high school students in China. As predicted, the level and type of parental support significantly impact the educational hope and life satisfaction of students.

It has been found that, on the one hand, intangible parental support, such as providing critical information, advice, encouragement, praise, and care, has a direct and significant impact on the well-being of young people. On the other hand, tangible parental support, which includes homework supervision, financial assistance, and educational resources, has a relatively less significant effect. This finding is consistent with previous research that suggests that positive parent–child relationships are beneficial for young people’s well-being (Shenaar-Golan and Goldberg, 2019; Koestner et al., 2020). A recent study (Yan et al., 2021) also found that the subjective well-being of Chinese high school students is closely linked to warm and caring relationships with their parents.

A positive parent–child relationship is conducive for children to feel like valuable members of their family who are loved and cared for. This creates a harmonious, happy, and loving family environment. According to SDT, when students have a good relationship with their parents or significant family members such as grandparents, they are more likely to feel a sense of belonging and connection to others, which can lead to better overall well-being (Ryan and Deci, 2000). Cultural factors also reinforce the impact of parents on children’s happiness. In Confucian heritage societies, the bond between parents and children is considered the core of the family (Fei, 2006) and a critical component of happiness (Gu, 2016). The family is at the center of youth development (Liang, 2005) and parent–child relationships are the prime indicator of life satisfaction among Chinese youth.

Our research findings confirm that intangible parental support, such as warmth, care, and knowledge exchange through conversations and interactions, directly promotes students’ educational aspirations (reflected in their desired highest educational level) and goal commitment (demonstrated by their efforts, appreciation, and agency in *gaokao*). This highlights the crucial role that families play in nurturing educational hope and pursuits. In other words, parents are significant figures who shape their children’s educational beliefs. This influence is deeply rooted in Chinese culture, where filial piety is a core familial value. Regardless of their family’s socioeconomic status, children are taught to respect and honor their parents by striving for academic excellence. Pursuing high *gaokao* scores is a way of showing respect to senior family members (Wang and Rao 2022). Therefore, for Chinese high school students, a positive parent–child relationship is essential as it enables parents to convey their perspectives and attitudes toward education, leading to a stronger sense of educational hope and a desire to pursue high-quality education in their children.

It is important to note that the impact of parental support on a child’s goal commitment varies among children from different educational backgrounds. The impact of parental support is more significant among children whose parents have a college education, highlighting the importance of family advantages (Lareau, 2000). The interaction between family members and people outside the family, such as relatives and friends, can influence how a child navigates their educational and social pathways (Bourdieu, 1977, 1986). Parents who have a low level of education may lack the confidence to get involved in their children’s education and are more likely to defer to teachers for advice, meaning that teachers and peers may influence their children’s educational hopes. On the other hand, parents with higher levels of education have more knowledge and information about education and are better equipped to encourage and provide guidance, thus, more readily playing a role in constructing their children’s educational hopes and planning for their future together with their children.

### 7.2 Educational hope: a complex construct

We can discern that educational hope is a complex concept, with different aspects exerting varying impacts on subjective well-being. Only goal commitment, which refers to the students’ proactive engagement and agency in pursuing educational goals, positively impacts life satisfaction. In contrast, educational aspirations do not positively predict satisfaction and have a significant negative predictive effect for students whose parents have a college experience.

On the one hand, goal commitment reflects individuals’ agency and the perseverance, determination, and effort put forth to achieve their objectives. Individuals with high commitment are those who more readily perceive the meaning in life and motivation, so they are likely to feel satisfied with their lives as they strive toward their aspirations (Guse and Shaw, 2018). Besides, the emphasis on education in Chinese culture further enhances the life satisfaction that students experience as a result of actively pursuing their goals. The ethics of diligent learning and self-cultivation (known as *xiu shen* in Chinese) is part of the work ethic embedded in Confucian values (Peow, 2015). From ancient times to the present, being diligent has been revered by Chinese and East Asians as a meaningful code of conduct and way of life. Therefore, it is natural that the active pursuit of educational goals would positively affect life satisfaction.

It has been found that having educational aspirations does not necessarily lead to increased well-being, which contradicts previous research (Cao et al., 2022). The reason for this could be that the previous research did not differentiate between different aspects of educational hope. In reality, the positive impact of educational hope on well-being is not related to the height of goal setting but rather whether it motivates individuals to strive toward meaningful goals and take action. It is important to note that for students whose parents have a college education, having high educational aspirations may even have a negative impact on their life satisfaction. When expectations are too high, they can lead to direct pressure on students. Additionally, parents with higher levels of education often have higher expectations for their children’s education. This is further compounded by Chinese culture, which emphasizes the importance of education in social class reproduction. The pursuit of excellence is often emphasized by families and schools to instill the hope of upward social mobility, career success, and social status (Liu and Helwig 2022). Well-educated parents with more knowledge of education are more likely to view access to higher education as an institutionalized channel for achieving future success (Kwon et al., 2017; Cheng et al., 2021).

### 7.3 Intangible parental support vs. tangible parental support

The university entrance exams in China, known as *gaokao*, involve many high school students, their families, and school staff. It is important to pay attention to the well-being of these students, especially to identify practical solutions that can help them aspire toward their desired educational pathway and increase their inner strengths. This study emphasizes that family support plays a crucial role in shaping Chinese youth’s educational goals, commitment, and overall well-being. Intangible parental support has more advantages than tangible support in shaping students’ attitudes toward education and their commitment to *gaokao*. Education is class-based and involves a process of class reproduction. Educated middle-class parents are better positioned to pass their university knowledge on to their children. Coleman (1994) argues that actors’ social actions are rational and that they tend to seek more resources. Information and advice directly contribute to realizing students’ interest in understanding the meaning of life, the value of education, and the importance of *gaokao*. Persuasive communication, encouragement, and praise gradually shape students’ aspirations and determination. They observe the long-term impact of intangible resources on their values, attitudes, autonomy, and self-agency.

In addition, the results verify that intangible parental support has a stronger impact on students whose parents have a college experience compared to those who do not. The research also notes differential intangible parental support. Educated parents provide more specific advice and support and are more knowledgeable in guiding their children toward making better decisions about their education. These parents are more aware of the importance of family education and spare no effort in cultivating their children’s educational aspirations (Lareau, 2000; Ho, 2010). Bourdieu’s family habitus theory suggests that a family’s sociocultural capital provides valuable intangible support. The slope of the positive linear relationship between intangible parental support and goal commitment is noticeable among students with educated parents. In other words, the impact of intangible parental support is strong among these students.

### 7.4 Major insights and implications

Our findings have highlighted the important role that parents play in the educational success and overall well-being of high school students. The implications of these findings are significant. Parents of students preparing for the *gaokao* examination, regardless of their own level of education, are advised to prioritize spending quality time with their children. Developing a strong parent–child relationship through personalized care, concern, and support is more beneficial than simply providing material resources, extra tutoring, or constant supervision. This approach will foster a sense of belonging and closeness and will inspire children to aspire toward higher education.

Parents should work collaboratively with their children to plan their growth and development instead of acting as directors. They should avoid imposing their own desires on their children, which can cause them to feel overwhelmed and anxious about the future. Instead, parents should co-create educational aspirations with their children that align with their development and guide them in actively pursuing these goals while encouraging their commitment to achieving them.

Research has shown that when parents increase their children’s enthusiasm for higher education, it develops their academic self-efficacy and helps them overcome educational obstacles. Parents can become friends with each other and provide additional support by hiring professional homework tutors to improve their children’s education, instead of relying solely on schools or after-school counseling institutions. It is important to collaborate with children in setting educational goals that align with their developmental trajectory and educational hope.

Goal commitment is important for students’ inner strength and can help them deal with the effects of intangible parental support on life satisfaction. Empowering students with positive support and advice to make informed educational choices in a supportive environment fosters a sense of personal investment in their growth and success. By understanding the role of functional families in connection with educational hope and life satisfaction, schools can tailor intervention programs to meet students’ specific needs. Positive parental support brings warmth, emotional closeness, and care, while school interventions can compensate for the lack of familial educational support among disadvantaged students.

## 8 Limitations

There are several limitations to our research that we need to acknowledge. Firstly, we used convenience sampling, which may lead to sampling bias. Our sample was drawn from one city in China due to difficulties in obtaining permission from schools and cities, especially during the strict social distancing regulations to curb the spread of the COVID-19 pandemic in China. This unique sociocultural context could have influenced the observed associations between the variables. Therefore, future studies should broaden the scope of this study to include other cities to deepen our understanding of the differences between cities in China.

Secondly, our sample size was small, which means that our findings are indicative rather than representative. The territory-wide pandemic interruptions undoubtedly affected the participants’ well-being and willingness to participate in the project. They could have been tired of attending online courses, and their exhaustion could have interfered with their learning and brought academic pressure to some degree. At the beginning of the resumption of face-to-face classes, high school students still suffered from stress and challenges brought on by the unique pandemic situation in China.

Thirdly, the cross-sectional research method we used may not be adequate to capture the changes in students’ well-being over time. Students’ assessment of well-being changes with time and social circumstances. Finally, the cross-sectional results’ causality is not sufficiently rigorous. Longitudinal studies are needed to track the changes in the differential impacts of different factors on *gaokao* students’ well-being and aspirations for higher education.

## Data availability statement

The datasets presented in this article are not readily available because the data involves school privacy concerns and the permissions do not allow the authors to disclose the original data. The data cannot be disclosed without the updated permission of the schools. Requests to access the datasets should be directed to hanfengjyy@mail.tsinghua.edu.cn.

## Ethics statement

The studies involving humans were approved by the ethics committee at the Chinese University of Hong Kong. The studies were conducted in accordance with the local legislation and institutional requirements. Written informed consent for participation in this study was provided by the participants' legal guardians/next of kin. Written informed consent was obtained from the individual(s), and minor(s)' legal guardian/next of kin, for the publication of any potentially identifiable images or data included in this article.

## Author contributions

FH: Conceptualization, Data curation, Formal analysis, Funding acquisition, Investigation, Methodology, Project administration, Resources, Validation, Writing – original draft, Writing – review & editing. CY: Conceptualization, Methodology, Supervision, Writing – review & editing.
